# Osteosynthesis of an intertrochanteric fracture on osteopetrosis A case report

**DOI:** 10.1016/j.ijscr.2024.109568

**Published:** 2024-03-20

**Authors:** K. Tabbak, M.A. Kharroube, F. Lamnaouar, C. Elkassimi, A. Rafaoui, M. Rafai

**Affiliations:** P32 CHU Ibn Rochd, Casablanca, Morocco

**Keywords:** Osteopetrosis, Marble bone disease, Albers–Schonberg disease, Atypical femur fracture, Erlenmeyer flask deformity, Osteosclerosis, Hyperostosis

## Abstract

**Introduction:**

Osteopetrosis is a rare hereditary disease that can be transmitted in an autosomal recessive or autosomal dominant.

**Case report:**

Here, we report a case of trochanteric fracture in an 18-year-old boy with an anatomical plate. At the last follow-up, 24 months after surgery, the fracture had healed well, and the patient was not restricted in his activities.

**Discussion:**

Osteopetrosis is a rare bone disease that is mainly caused by osteoclast dysfunction. It results from a remodelling defect that leads to hypermineralization of the skeleton, resulting in bone fragility. Both surgical and nonsurgical management have advantages and disadvantages. Thus, open reduction and anatomic plate fixation remain effective management modalities for trochanteric fractures in osteopetrosis patients.

**Conclusion:**

For our patient and as described in the literature, the complication rate decreases as some principles are respected with better consolidation of the osteoporotic fracture.

## Highlights

• Osteopetrosis due to osteoclast dysfunction. result is a remodeling defect that leads to hypermineralization of the skeleton, resulting in bone fragility.
• Operative intervention, presents many technical challenges
• some principles are to respect in the osteosynthesis of fractures in osteopetrotic bone to avoid complications

## 1 Introduction

Osteopetrosis is a rare bone disease that is mainly caused by osteoclast dysfunction. It results from a remodelling defect that leads to hypermineralization of the skeleton, resulting in bone fragility (often multiple pathological fractures), compression of the cranial nerves (optic atrophy, etc.), osteomyelitis and smothering of the hematopoietic marrow, which can progress to pancytopenia [1].

There are different forms of osteopetrosis, with a variable course ranging from death in utero to a completely asymptomatic form [2].

There is no satisfactory treatment for osteopetrosis. In the malignant form, bone marrow transplantation can, in favourable cases, halt progression and restore functional bone remodelling [3].

We report a case of a trochanteric fracture in an 18-year-old boy treated with a locking plate for the proximal femur, with a highly satisfactory functional outcome after one year.

## 2 Case report

The reporting of this work followed the SCARE checklist criteria [12], ensuring adherence to guidelines for quality reporting in case series.

We admitted a patient 18 years old with no history of a fracture who suffered a “closed” trauma of the left hip following a sports accident.

Clinically, the patient presented total functional disability, with pain in his left thigh and no downstream vascular-nervous disorders.

**Fig. 1:**
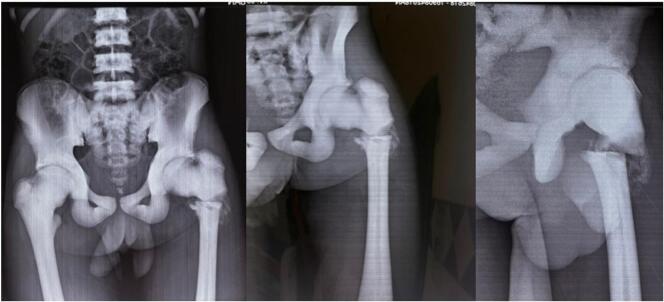
Radiological aspect of the atypical fracture: transverse fracture line with no comminution, lateral thickening of the periosteum, generalized increase in cortical thickness of the femoral diaphysis.

The patient underwent a radiological examination, which revealed a left trochanteric fracture on an osteosclerotic (Fig. 1) bone with features seen in atypical femoral fractures:

- transverse fracture line with no comminution
- Lateral thickening of the periosteum
- Generalized increase in cortical thickness of the femoral diaphysis

**Fig. 2:**
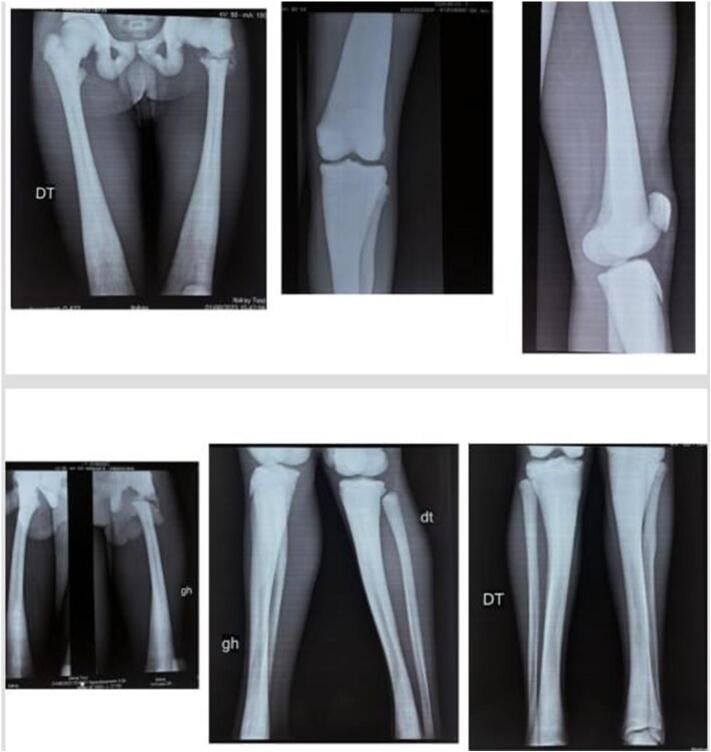
Diffuse osteosclerosis (trabecular bone) and hyperostosis (cortical bone) of the appendicular skeleton segments, an Erlenmeyer deformity (metaphysical enlargement), and rugger jersey (sandwich) spine.

Radiographs of the patient's bone segments revealed generalized osteosclerosis, an Erlenmeyer Flask Bone Deformity (without the alternating sclerotic band observed in Dysosteosclerosis), and a rugger jersey spine (Figs. 2).

We were in front of a bone disorder with an atypical femur fracture, and we started again with a medical interview and clinical examination. We did not find a known similar case in the family. The mother died two years ago and was shorter than her son, and the father was taller with a normal skeleton according to X-ray images. Additionally, the clinical exam did not reveal dysmorphism or mental retardation signs or dental anomalies, and there was no sign of cranial nerve compression with loss of visual or hearing acuity. Blood screening was normal, with no signs of pancytopenia or renal dysfunction.

We were probably in front of a benign form of osteopetrosis. We could not perform a genetic study to determine genetic abnormalities.

After reviewing the literature, we decided on surgical treatment using a locking plate on the proximal end of the femur.

The planning for the surgery included the use of a slow drilling motor and multiple drill bits.

Under general anaesthesia, the patient was placed in a lateral decubitus position supported by 2 pubic and sacral supports. An incision was made along an imaginary line between the lateral femoral epicondyle and the greater trochanter. After opening the fascia lata, the fibres at the origin of the vastus lateralis were elevated from the intermuscular septum, and the vastus lateralis was separated by blunt dissection from the fascia lata. The vastus lateralis retracted anteromedially. The muscle fascia investing the vastus lateralis is incised approximately 1 cm anterior to the intermuscular septum.

**Fig. 3:**
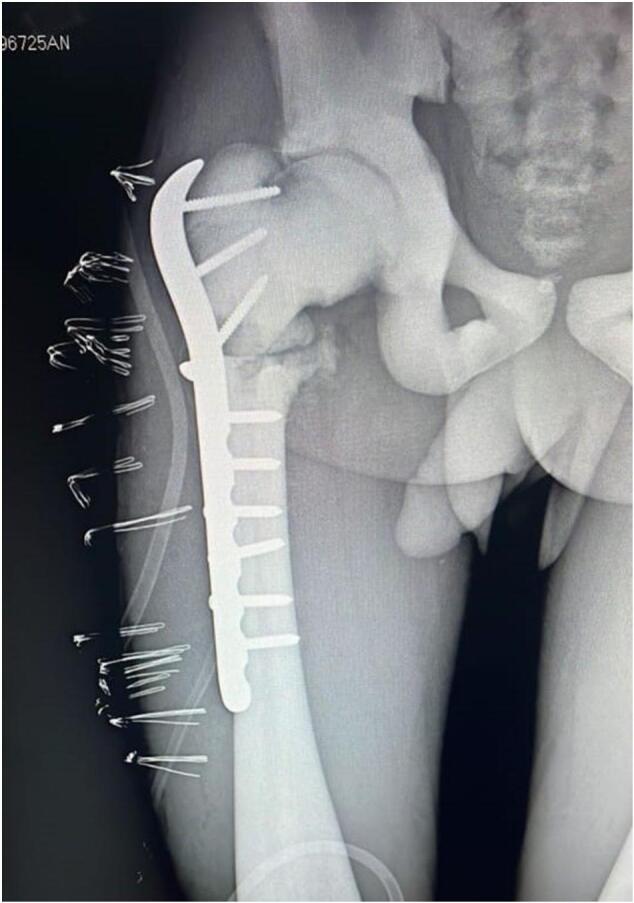
Postoperative X-rays of the left hip.

We identified the fracture site, which we prepared and reduced with forceps, and placed an anatomical plate and screws. The path of the screws was particularly difficult to drill due to bone sclerosis. We prepared multiple drill bits, and while drilling, we performed continuous irrigation with saline. A post-operative x-rays was realised (Fig. 3).

Antibiotic coverage was maintained for two days, and there were no local complications.

The patient was authorized to verticalize on day 1 after surgery, partial loading was applied after the fourth week, and total loading was applied after the second month.

**Fig. 4:**
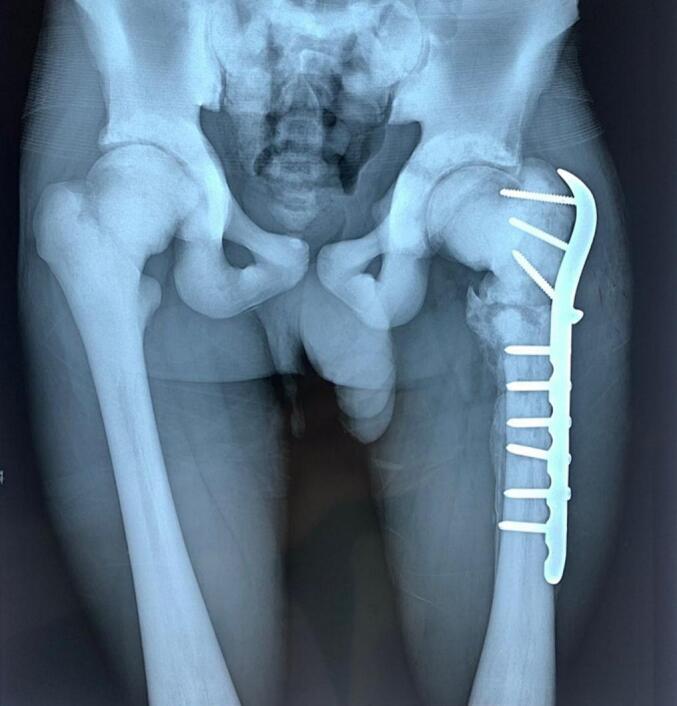
Six months after osteosynthesis, a satisfactory callus was observed along the plate height and the fracture site.

The patient was regularly reviewed via consultation, and the X-rays at 6 months showed a good periosteal callus (Fig. 4).

## 3 Discussion

The name osteopetrosis is derived from the Greek language. ‘Osteo’ means bone, and ‘petrosis’ means stone. Therefore, the disease is often referred to colloquially as “marble bone disease.” The disease was originally described by a radiologist in Germany, Dr. Albers-Schonberg, in 1904. Bone with abnormally increased density is the key radiographic finding. This increased density is secondary to osteoclast dysfunction and leads to the affected bones being abnormally brittle [4].

Osteopetrosis is a rare group of bone disorders characterized by osteoclast dysfunction that causes an increase in bone density, impaired remodelling, and thus bone fragility. Intraosteoclast deficiency of the enzyme carbonic anhydrase, as well as at least 10 gene mutations—most importantly, the chloride channel gene CLCN7—have been associated with this disease. Patterns include an autosomal recessive form, osteopetrosis congenita, which carries a poor prognosis with death, usually in infancy. The autosomal dominant form, osteopetrosis tarda, is usually asymptomatic and is found in adults. Malignant variants, known as marble bone disease, are diagnosed in childhood and manifest as recurrent bony fractures, short stature, skull thickening, sensorineural hearing loss, psychomotor retardation, and distal renal tubular acidosis. Bony pain, osteomyelitis, and degenerative joint disease frequently occur [5].

Healing and fracture remodelling in osteopetrotic patients are unpredictable. The healing response is variable [6].

The histologic features of bone callus after a traumatic fracture in a patient with osteopetrosis are presented. The fracture callus develops in stages that are apparently normal. The tissue is initially rich in bone-forming cells and vessels. One year later, however, unlike mature osteopetrotic bone, the tissue shows no Haversian organization [7].

Management must be individualized. Decisions on whether to operate, mobilize, and allow normal daily activities must be made on a case-by-case basis, as there are no fixed guidelines for management. An informed decision must be made regarding fracture management; the surgeon should always have a backup plan if the initial option fails [6].

A review of the available literature showed that conservative treatments can be successful [[5], [6], [7], [8]].

Operative intervention presents many technical challenges. Hard bone may be penetrated with a drill, resulting in high friction, and prolonged drilling can blunt or break the drill bit. In addition, because of the difficulties encountered during the operation, the operative time may be prolonged, thereby increasing the risk of postoperative infection. There is also a risk of delayed consolidation and nonunion due to impaired bone remodelling [8].

[Ding et al] reviewed 41 case reports and small-scale case reports, including 6 cases of femoral neck fractures, 3 cases of femoral shaft fractures, and 32 cases of pertrochanteric fractures. In a total of 50 operations, there was a 6.00 % nonunion rate and a 6.00 % infection rate, and all infected fractures failed to achieve union. In this cohort, the rate of hardware failure was 16.00 %, and the incidence of periprosthetic fracture was 6.00 %. Since Ding et al. reported that occlusion of the medullary cavity and femoral malformation limited the application of intramedullary fixation, DHS fixation was used. At the same time, two sets of surgical instruments were prepared for use alternately, along with sterile ice-cold saline irrigation for cooling down, to solve the problem of overheating the drill, which aided the successful completion of the operation [9] (Table 1).

**Table 1:** Ding et al. [9] difficulties during osteosynthesis in osteopetrotic bone and the different solutions.

Challenges	Solutions
- Drilling skills - Intraoperative fracture - Temperature control - Infection - Hip lag screw - Lateral plate	Spaced cycles with low-speed drilling or use high resistance and high speed electric drills bits Avoid inappropriate violence and use of hammers Continuous cooling with saline, frequent change of drill bits Strict aseptic operation, control of operation time, preventive use of drug as necessary. Reducing length of drilling, tapping and inserting screws, regular cleaning of tap and screw tract Fully tapping all hales before screw insertion

Technical difficulties include the bending of drill bits or screws during surgery using drilling or carving due to hard but fragile sclerotic bones and a narrow medullary canal. Slow-speed high-torque electric drills, as well as frequent cooling with physiological saline, clearance of drill grooves, and the use of a staggered drill system, have been recommended. However, there is still an increased risk of implant failure and nonunion for internal fixation. To avoid such complications, some authors have recommended bone morphogenic protein (BMP) grafting, which stimulates mesenchymal cells and differentiation into osteoblasts due to its osteoinductive nature, thereby exerting a positive effect [10].

Dawar et al. [11] proposed several principles for the osteosynthesis of fractures in osteopetrotic patients:

- There is no need for compression of the fracture site, and a secondary union is preferred.
- Intramedullary implants are too difficult to insert, and an extramedullary implant is preferred.
- Always prefer a locking plate
- Prepare multiple drill bits, perform frequent cleaning of the drill bit of bone, and avoid any toggle
- To avoid osteonecrosis while drilling, continuous irrigation with saline should be performed.
- Use a tap before the insertion of a cortical screw, avoid screw damage and always check for good size (difficult to exchange the screw).
- Avoid the use of cancellous screws
- To ensure stress distribution, the last screw must be uni-cortical or inserted at an obtuse angle.
- Avoid, as much as possible, implant removal
- Given the impaired vascularity and hampered immunity, there must be appropriate antibiotic coverage and reduced surgical, time, and respect for soft tissue during dissection and fixation.
- For total hip arthroplasty, uncemented arthroplasty is preferred; for total knee arthroplasty, alignment is assured in the extramedullary region.

## 4 Conclusion

Osteopetrosis remains a rare condition that can be complicated by fractures, as in our case.

There is currently no consensus on the optimal treatment method for trochanteric fractures in osteopetrosis patients.

Surgical treatment remains an important option, despite the difficulties posed by this condition and the relatively high rate of complications compared with a similar fracture in healthy bone.

## CRediT authorship contribution statement

All the authors contributed to the study concept, data analysis and writing of the paper.

## Funding

This research did not receive any specific grant from funding agencies in the public, commercial, or not-for-profit sectors.

## Ethical approval

The case report is exempt from ethical approval at our institution, and only consent is necessary.

## Consent

Written informed consent was obtained from the patient for publication of this case report and accompanying images.

## Declaration of competing interest

The authors declare no conflicts of interest.
